# Gleanings in Science and Medicine

**Published:** 1893-08-26

**Authors:** 


### Gleanings in Science and Medicine.

Soot now is added to the already lengthy existing list of substances which act as disinfectants. Charcoal has long been recognised as possessing purifying qualities, and soot, which is a like element in a different form, has a great power of absorbing foul gases and clearing the surrounding atmosphere. It certainly is an economical and easily-obtained disinfectant, and if freely sprinkled about acts in a quick and effective manner.

A Peruvian has just patented an invention by which the speed of any vessel can be immediately checked at sea. The expedient is a simple enough one, and it seems almost strange that it has not occurred sooner to any practical mind. The patent takes the form of a sliding frame, which is placed on a post at the bows of a vessel having pivoted wings on its side, which offer resistance, transversely, to the forward motion of the ship when this frame is in its lowermost position.

From Glasgow we learn that Dr. Hugh Thompson describes nine cases in which he inoculated children with fresh serum, extracted from patients who were suffering from measles. Slight catarrhal and other symptoms followed on the point of insertion, the operation only failing in two cases out of the nine. The serum, which was taken from the infected persons, was inserted immediately into the children on whom the experiment was being tried, and the *modus operandi* was through superficial scarifications.

For a history of dentistry in an abridged and condensed form we are indebted to an article appearing in a current American monthly, and which throws much light on the subject. From the earliest times we find how mention is made of the care of teeth; 500 years B.C. Herodotus, writing of his travels in Egypt, mentions divisions of medicine being made in that country under the following branches: "Some physicians," he says, "are for eyes, some for the head, others for teeth, and others for internal disorders." Whilst in the heads of mummies of about this period gold fittings have been found in perfect repair. This primitive dentistry is historically instructive of the strides which science has made since that period. When it thus first occurred to man to supplement artificial for natural teeth an inspection of the heads of mummies who had thus been treated during lifetime reveals that the first teeth thus substituted were fastened into the mouth by ligatures of gold wire bound on to neighbouring ones, in what must have been a most inconvenient method for the person thus doctored. Ancient Greece was long called "The nursery of modern medicine," and here a special study of dentistry was encouraged, and alluded to by Aristotle, 300 B.C.

The possible disadvantages which exist in transplanting animal life from one climate to another is pointed out in the pages of a weekly contemporary. The case herein mentioned is that of a Frenchman, who twenty years ago, tried to introduce the silkworm into Massachusetts with a view to cultivation on an increased scale, in what appeared to the experimenter to be a favourable climate for his project. A handful of the worms were blown by accident from the owner's keeping into a neighbouring yard, where they remained, failing to be discovered. Later on, the surrounding country became overwhelmed by a plague of gypsy moths, whose ravages were of such a nature as to compare, on equal terms, with the desolation wrought by fire and flood. This was followed by a complete plague of the moths, and for five years the Agricultural Department of Massachusetts has failed to exterminate the demolishing invader. These pests have now spread themselves over two hundred miles of country, and the State has voted £25,000 for the purpose of securing their destruction. The writer who brings these facts before the English press concludes his remarks by pointing out that "it would seem that the balance of Nature, through the transplanting of those silkworms has been considerably interfered with."

In spite of fears entertained in certain quarters, we are glad to learn that the faculty of the John Hopkins Medical School see their way to the opening of the school on October 2nd, and the institution, according to Miss Garrett's gift, is to be thrown open to both sexes alike.

It is being urged that each railway should have its surgeon, whose existence is as much a necessity as its directorate. The promoters of the scheme have not enlarged upon it farther than the primary suggestion, nor do they explain how one surgeon could take into his practise an area rather larger than the most ambitious of mortals could contemplate. Should the company decide, for instance, that their railway surgeon's headquarters were to be fixed in London, he could barely be of any first aid to the wounded were an accident to occur at Edinburgh. But then, perhaps, each train is to carry its own "cure," and if so, the plethora of young English doctors may rejoice at having a prospect of unlimited practise shortly thrown open to them.

The Middle Ages saw much progress made in the art; histories were written and books published on the subject, treating dentistry from an anatomical and physiological aspect, to be superseded later on by the more definitely practical form which it has now assumed. No country in modern times has made greater advances in this direction than America, to which people the care of their teeth is an inherent law in their nature. Indeed, the dentist's bill is a substantial item in the accounts of our transatlantic cousins. To-day, no fewer than three colleges exist for dentistry in America, whilst equal with the increase of facilities to the study of the art is the gigantic demand for dental mechanism and materials. One house alone devoted to this manufacture has six provincial branches, and gives employment to 1,200 hands.

Paris is now looking to its laurels as regards its water supply, a considerable quantity having been recently introduced into the city, which bids fair to promise a greater immunity from typhoid-fever breeding properties than has hitherto been the case with the water of Paris. Added to this advance movement in the hygiene of the French capital is the additional overhauling of the ice supply resources. It appears that the local supply has, up to the present time, been dependent on such sources as the Epinay-Enghian basin, a pool connected with the River Seine, and a receptacle, *pur et simple*, for the sewage of the city. Dr. Prudden's laborious investigations have now satisfied the public mind that water does not lose any of its original harmful character through the process of freezing; if it was impure as water, it becomes little less so as ice, and therefore it is equally important to inquire more strictly into the sources of both supplies.

The sight of the ordinary brass thimble is a familiar one to our eyes. Every working woman of our acquaintance possesses such an one; it is either on her finger, or temporarily at rest in her pocket. The feminine sewing world in general has grown up to regard this necessary protection against the needle's point as a friend, and not as a foe; but, alas! we are now brought face to face with facts, and we learn, with some consternation, that there lurks a danger in the thimble's brassy depths. One such has lately acted the part of poisoner upon its owner. It appears that the woman had a slight scratch on the finger, on which she placed the thimble, and commenced some sewing. Ere night she had succumbed to blood poisoning. It is fortunate that more of the needle-working world have not ere this fallen victims to their thimbles, for the risk thus run of using corroded brass is no slight one. We are very apt not to allow the experiences of others to apply in our own cases, but it would be well if women would bear this tragic death in mind, and would substitute the bone thimble or the more dangerous one of brass.

